# Hereditary Bilateral Retinoblastoma Detected on Screening in a Two-Month-Old Infant With a Strong Family History

**DOI:** 10.7759/cureus.107302

**Published:** 2026-04-18

**Authors:** Daniel E Bhola, Ronnie M Bhola, Kevon Dindial

**Affiliations:** 1 Ophthalmology, Caribbean Vitreous and Retina Surgery Ltd/Trinidad Eye Hospital, San Fernando, TTO; 2 Pediatrics, Eric Williams Medical Sciences Complex, Champ Fleurs, TTO

**Keywords:** bilateral retinoblastoma, hereditary retinoblastoma, paediatric ophthalmology, retinoblastoma, screening

## Abstract

Retinoblastoma is the most common primary intraocular malignancy of childhood and may occur sporadically or as a heritable disease related to pathogenic variants in RB1. Early ophthalmic screening of at-risk infants can identify tumors at a stage amenable to globe-sparing therapy. We report a two-month-old asymptomatic female infant with a strong maternal family history of retinoblastoma who was found on screening to have a Group B lesion in the right eye and three Group A lesions in the left eye, according to the International Classification of Retinoblastoma. Following multidisciplinary evaluation, she underwent focal therapy with cryotherapy to the left eye and argon laser photocoagulation to the right eye, together with four 21-day cycles of systemic carboplatin, etoposide, and vincristine. Treatment was well tolerated with minimal reported adverse effects. This case highlights the importance of family history, early screening, and multidisciplinary care in children with suspected heritable retinoblastoma.

## Introduction

Retinoblastoma is the most common primary intraocular malignancy of childhood and arises following biallelic inactivation of the RB1 tumor suppressor gene [1,2]. It is a rare but vision- and life-threatening malignancy that typically presents before five years of age. Retinoblastoma may occur in sporadic or hereditary forms, with hereditary disease more commonly presenting at an earlier age with bilateral or multifocal tumors [1,2]. Due to the rarity of retinoblastoma, individual case reports remain useful in illustrating clinically important presentations, particularly when diagnosis occurs through screening in high-risk infants.

Recognition of heritable retinoblastoma is clinically important because it affects ocular management, family counselling, surveillance strategies, and long-term follow-up [2]. In hereditary retinoblastoma, a germline RB1 pathogenic variant is present from conception, which helps explain why the disease often presents earlier and may be bilateral or multifocal rather than unilateral [2]. Children with a positive family history are at increased risk and should undergo early ophthalmic surveillance, with genetic counselling and testing considered when appropriate to refine risk assessment and guide screening schedules [2,3]. Early diagnosis is critical because timely treatment improves survival and increases the likelihood of globe and vision preservation [1,4]. With current risk-stratified screening protocols and advances in globe-sparing treatment, early identification of heritable disease has become increasingly important in clinical practice [3,4]. This case is notable because retinoblastoma was detected on screening in an asymptomatic two-month-old infant with a striking maternal family history and bilateral disease at presentation. It adds to the existing literature by demonstrating the practical value of family history-based surveillance in enabling diagnosis at an early stage, allowing prompt multidisciplinary management and globe-sparing treatment in suspected hereditary retinoblastoma.

## Case presentation

**Figure 1:**
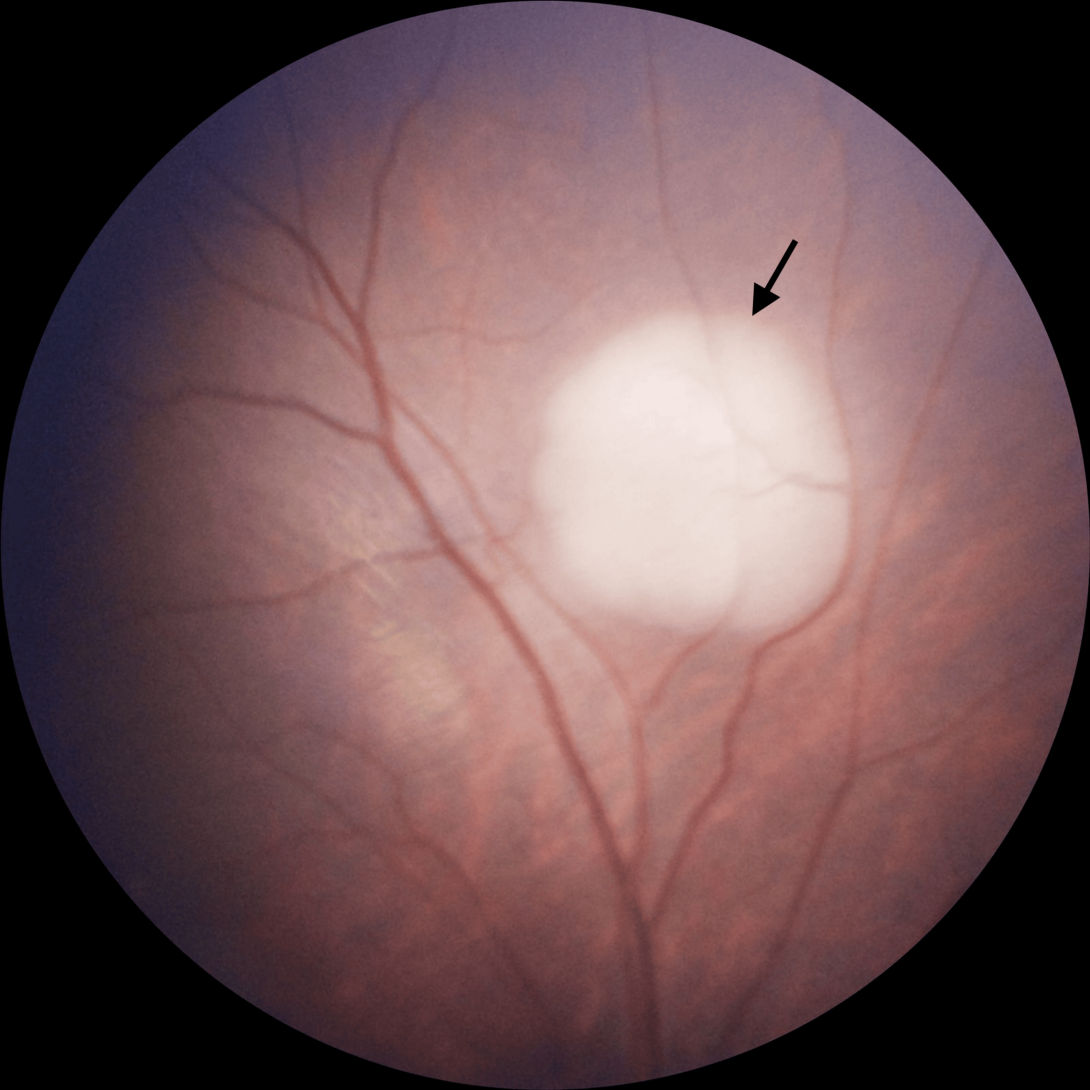
Pretreatment fundoscopic image of the right eye demonstrating a 3 mm elevated lesion located 1 mm from the superior arcade (arrow), consistent with Group B retinoblastoma

A two-month-old asymptomatic female infant underwent retinoblastoma screening due to a strong family history of the disease. Her mother had been diagnosed with retinoblastoma in childhood and underwent right enucleation. Of the patient’s three maternal uncles, two were also diagnosed with retinoblastoma; one survived, and the other died from complications of the disease. Fundus biomicroscopy of the right eye demonstrated a 3 mm elevated lesion located 1 mm from the superior arcade (Figure 1), consistent with Group B retinoblastoma according to the International Classification of Retinoblastoma [5]. Examination of the left eye revealed three mid-peripheral lesions, which were classified as Group A retinoblastoma [5]. MRI of the brain and orbits performed at initial staging showed no orbital mass, optic nerve abnormality, or intracranial involvement. 

**Figure 2:**
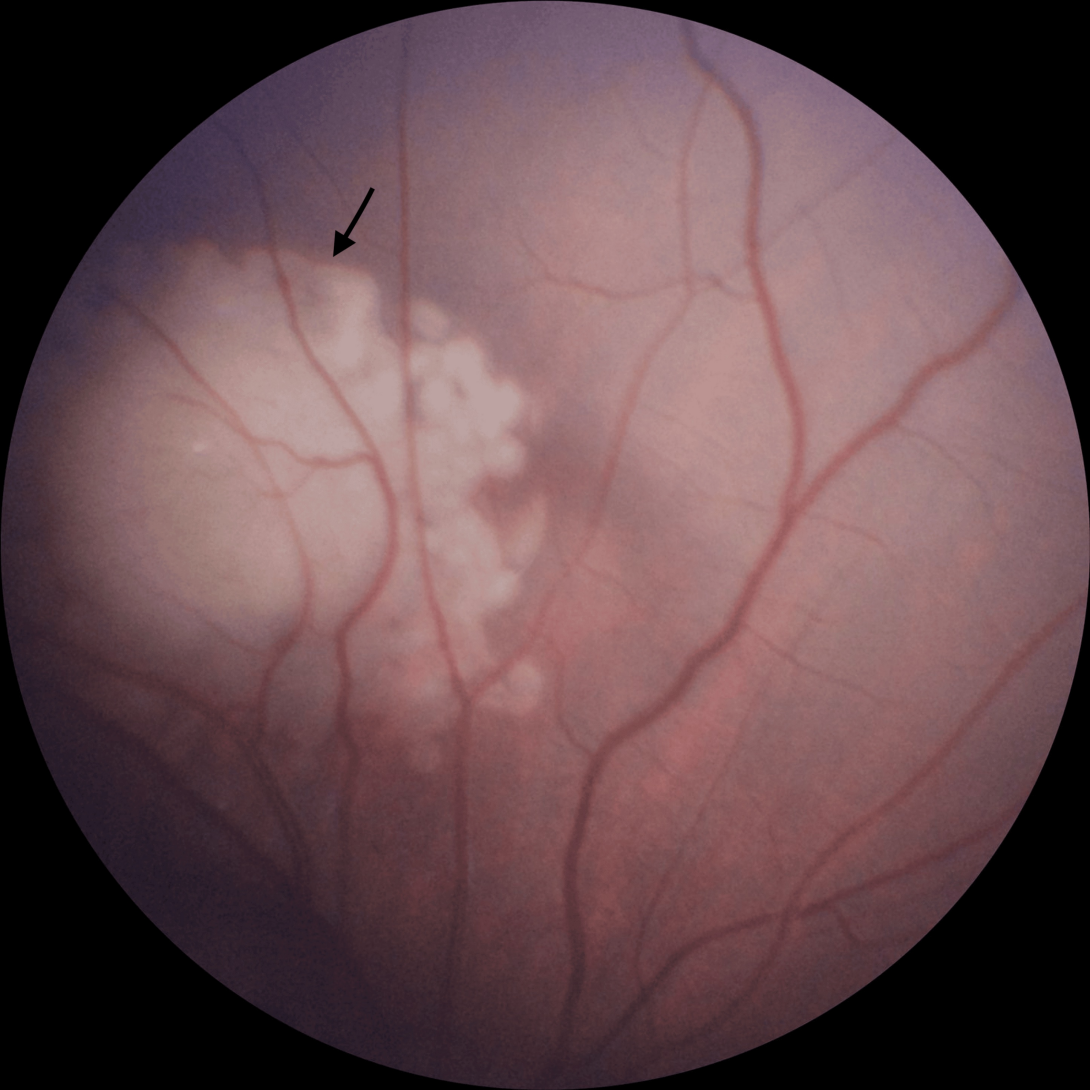
Post-treatment fundoscopic image of the right eye following argon laser photocoagulation (arrow)

Management was planned after a multidisciplinary discussion between the ophthalmologist and pediatric hematologist/oncologist. Given the posterior location of the right eye lesion, the patient underwent argon laser photocoagulation to the right eye, with the post-treatment appearance shown in Figure 2. The three peripheral lesions in the left eye were treated with cryotherapy. Following focal therapy, the patient received systemic chemotherapy following the Children's Oncology Group of North America standard of care protocol, consisting of four 21-day cycles of carboplatin, etoposide, and vincristine administered through a central line. Granulocyte colony-stimulating factor (G-CSF) was given to expedite neutrophil recovery.

Treatment was well tolerated, with minimal reported adverse effects. Surveillance MRI of the brain and orbits performed approximately one year later showed no evidence of recurrent disease, orbital masses, or intracranial findings. Early detection through screening allowed timely globe-sparing treatment. A summary of the key clinical findings, management, and follow-up outcome is provided in Table 1.

**Table 1:** Summary of clinical findings, management, and follow-up outcomes

Domain	Details
Age at presentation	Two months
Sex	Female
Presentation	Asymptomatic infant screened due to a strong family history of retinoblastoma
Family history	Mother diagnosed with retinoblastoma in childhood and treated with right enucleation; two of three maternal uncles were also affected
Laterality	Bilateral
Right eye findings	3 mm elevated lesion located 1 mm from the superior arcade
Right eye classification	Group B retinoblastoma
Left eye findings	Three mid-peripheral lesions
Left eye classification	Group A retinoblastoma
Initial staging imaging	MRI brain and orbits showed no orbital mass, optic nerve abnormality, or intracranial involvement
Initial management planning	Multidisciplinary discussion between ophthalmologist and pediatric hematologist/oncologist
Right eye treatment	Argon laser photocoagulation
Left eye treatment	Cryotherapy
Systemic treatment	Four 21-day cycles of carboplatin, etoposide, and vincristine
Supportive treatment	Granulocyte colony-stimulating factor (G-CSF)
Follow-up imaging	Surveillance MRI brain and orbits performed approximately one year later showed no evidence of recurrent disease, orbital masses, or intracranial findings
Follow-up outcome	Treatment was well tolerated with minimal reported adverse effects; surveillance imaging showed no evidence of recurrence
Clinical significance	Early screening enabled timely globe-sparing treatment in a high-risk infant

## Discussion

Retinoblastoma is the most common primary intraocular malignancy of childhood and typically presents before five years of age [1]. It may occur in either sporadic or hereditary forms, with hereditary retinoblastoma associated with pathogenic variants in the RB1 tumor suppressor gene and more likely to present at an earlier age with bilateral or multifocal disease [2]. In the present case, the patient was diagnosed at two months of age and had bilateral tumors, findings that strongly support a hereditary form of retinoblastoma.

**Figure 3:**
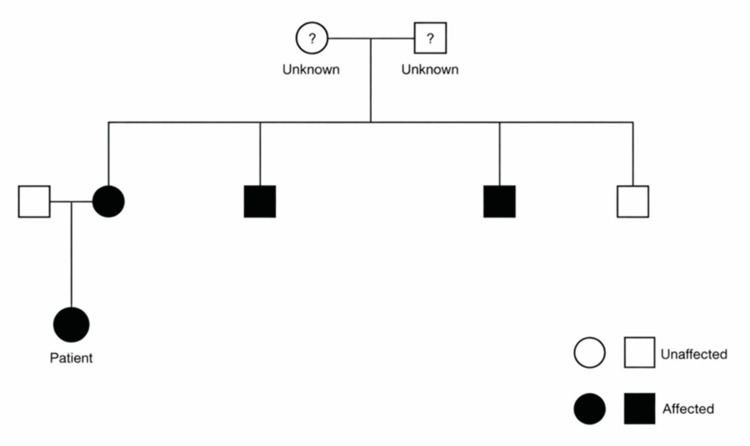
Pedigree demonstrating the maternal family history of retinoblastoma, supporting a suspected heritable disease Image Credit: Daniel E. Bhola.

The family history in this case is particularly significant. The patient’s mother had retinoblastoma in childhood and underwent right enucleation, and two of the three maternal uncles were also affected. The pedigree demonstrates clustering of disease across the maternal line, further supporting suspected heritable retinoblastoma (Figure 3). Formal RB1 genetic testing was not available in this case. However, the combination of bilateral presentation and a strongly positive family history makes the presence of a germline RB1 pathogenic variant highly likely [2]. This has important implications not only for the patient, but also for genetic counselling, future surveillance, and screening of other at-risk family members. Based on the highly likely premise that this patient has the RB1 mutation, she is also at risk for other malignancies such as osteosarcoma and pineoblastoma [2].

The International Classification of Retinoblastoma was useful in guiding management in this case [5]. The left eye lesions were classified as Group A and were treated with cryotherapy, while the right eye lesion was classified as Group B and managed with argon laser photocoagulation and systemic chemotherapy. This approach is consistent with the principle of tailoring treatment to tumor size, location, and extent, with the goal of achieving tumor control while preserving the globe whenever possible. In this patient, the posterior location of the right eye lesion made laser therapy an appropriate focal treatment option, while systemic chemotherapy provided additional control for bilateral disease. Management in this case was broadly consistent with established globe-sparing approaches for early intraocular retinoblastoma. Group A lesions are commonly amenable to focal therapies such as cryotherapy or laser photocoagulation, while Group B disease may also be managed with focal treatment with or without chemoreduction depending on tumor location, laterality, and overall disease burden [1,5]. In the present case, the bilateral early-stage presentation and posterior location of the right eye lesion supported the use of cryotherapy, argon laser photocoagulation, and systemic chemotherapy as an eye-preserving strategy. MRI performed at initial staging showed no orbital mass, optic nerve abnormality, or intracranial involvement, supporting the classification of early intraocular disease at presentation.

This case also highlights the importance of screening in children at increased risk for retinoblastoma. Individuals at high risk include those with a positive family history of retinoblastoma, and current screening recommendations emphasize early ophthalmologic assessment in at-risk children, with surveillance tailored to the likelihood of a germline RB1 mutation [2,3]. Early diagnosis has a major impact on clinical outcomes, with improved survival and greater potential for eye and vision preservation when tumors are detected before advanced progression [4]. This is reflected in published globe-salvage outcomes, with one long-term series reporting salvage rates of 96% for Group A eyes and 91% for Group B eyes, compared with lower rates in more advanced disease [6]. The patient in this report was asymptomatic at presentation, and the disease was detected only because screening was performed due to the known family history.

The clinical value of this case lies in demonstrating how appropriate surveillance can change outcomes. Because the tumors were identified early, the patient was managed with globe-sparing therapy rather than enucleation. The successful use of focal treatment together with systemic chemotherapy, along with minimal reported adverse effects, supports the benefit of early recognition and coordinated multidisciplinary care. Surveillance MRI performed approximately one year later showed no evidence of recurrent disease, orbital masses, or intracranial findings. Overall, this case reinforces the importance of family history, early referral, and structured screening in the management of children at risk for hereditary retinoblastoma.

## Conclusions

This case demonstrates that retinoblastoma may be detected on screening before the onset of symptoms in infants with a strong family history of the disease. In this patient, screening identified bilateral tumors at an early stage, allowing globe-sparing treatment with argon laser photocoagulation, cryotherapy, and systemic chemotherapy. Initial staging MRI showed no orbital or intracranial involvement, and surveillance MRI performed approximately one year later showed no evidence of recurrent disease. This case reinforces the practical value of early referral, careful family history assessment, and multidisciplinary management in suspected hereditary retinoblastoma.
